# Beyond headcount: four dimensions of Canada’s primary care access crisis and a three-level agenda for action

**DOI:** 10.3389/fmed.2025.1695409

**Published:** 2025-11-20

**Authors:** Maisam Najafizada

**Affiliations:** Division of Population Health and Applied Health Sciences, Faculty of Medicine, Memorial University of Newfoundland, St. John’s, NL, Canada

**Keywords:** primary care (PC), access, family physician (FP), family physician shortage, Canada

## Abstract

Public debate in Canada often diagnoses a simple “shortage of family physicians,” yet system indicators point to a more complex access problem. In 2023, 17% of adults reported no regular primary care provider, only 26% obtained same/next-day appointments, and about 15% of emergency department visits were potentially primary-care-manageable—over half potentially manageable virtually. Meanwhile, average weekly physician work hours have declined by 6.9 h since the late 1980’s and the average number of patients seen per family physician fell from 1,746 (2013) to 1,353 (2021), alongside a shift away from comprehensive community practice. Drawing on comparative evidence that stronger primary care architecture is associated with better performance and that primary health care averages ∼13% of current health spending across OECD countries, this Perspective reframes Canada’s challenge across four dimensions: effective capacity (not just headcount); demand—complexity, time, and continuity; maldistribution and loss of comprehensive care; and system entry-point design. We then organize solutions in three groups: system-level (investment floors, enrollment/rostering and after-hours obligations, payment aligned to continuity and team-based comprehensiveness), organizational-level (interdisciplinary teams, task-sharing with NPs/pharmacists/PAs, operationalized continuity), and data & research (effective-FTE and continuity metrics, complexity-adjusted panel targets, rigorous evaluation of entry-point and scope reforms). Recasting the problem from headcount to capacity-and-design clarifies actionable levers for timely attachment and sustained relational continuity.

## 1 Introduction

Public debate in Canada often reduces primary care access problems to a simple “shortage of family physicians (FPs).” Yet system monitoring shows persistent access gaps: in 2023, 17% of adults reported no regular primary care provider; only 26% reported same- or next-day appointments; and 15% of emergency department visits (April 2023–March 2024) were for conditions potentially manageable in primary care, over half of which could potentially have been managed virtually (1). These indicators suggest a complex access problem that is experienced by the public as “shortage.”

At the same time, supply-side signals complicate a headcount narrative. Average weekly physician work hours have declined by 6.9 h since the late 1980’s (2), and the average number of patients seen per FP per year fell from 1,746 in 2013 to 1,353 in 2021 (3). In parallel, a growing share of physicians practice outside traditional community-based primary care (4). Together, these patterns indicate that the public’s experience of “shortage” reflects multiple interacting dynamics rather than a single deficit in headcount.

Comparative evidence underscores different dimensions access to primary care. Across OECD countries, primary health care averages about 13% of current health spending, and stronger primary care architecture is linked to better access and system performance (5). Canada’s access metrics point to a need to reconsider how we define and target the problem the public experiences as “shortage.”

**FIGURE 1:**
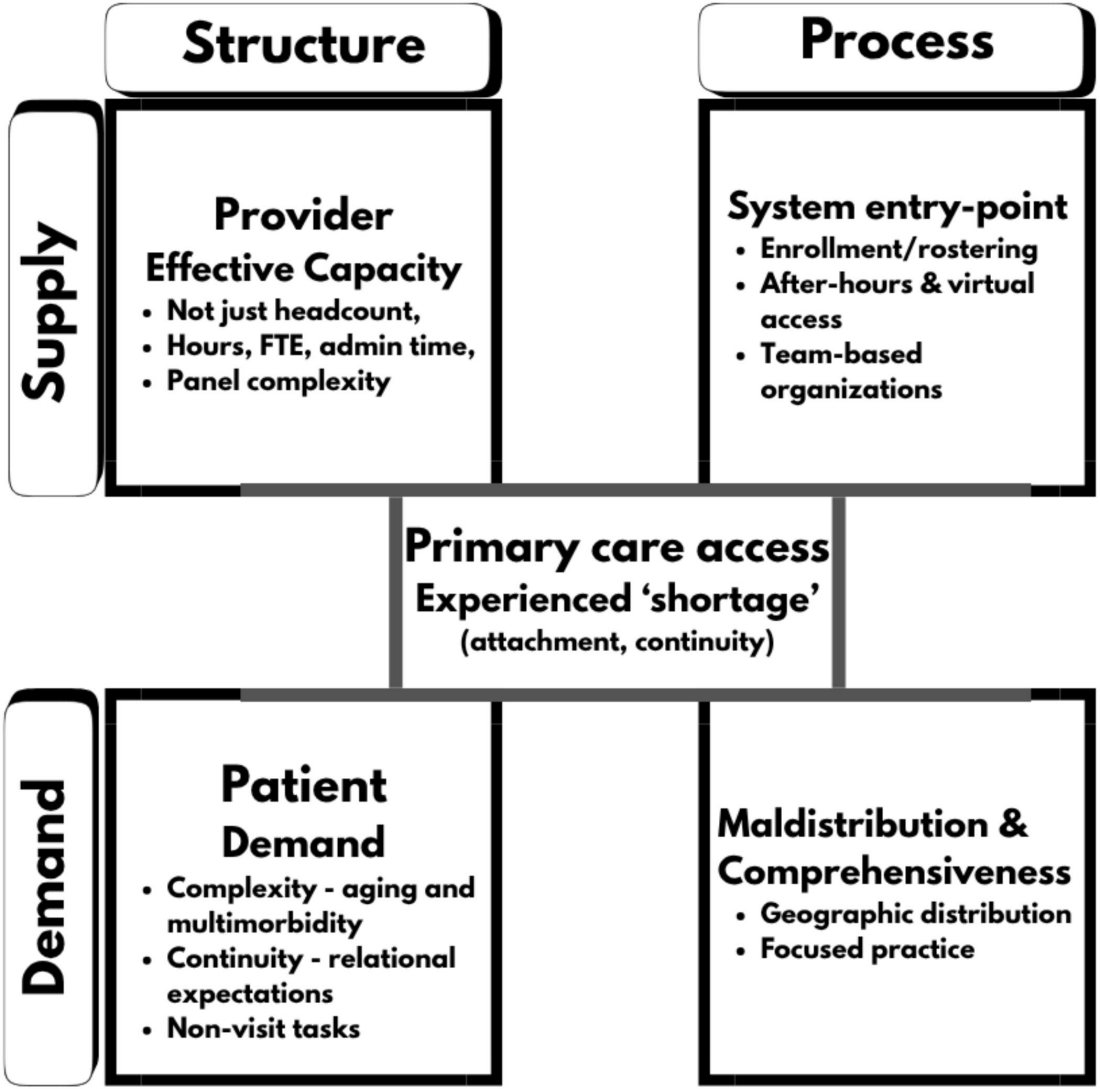
Four dimensions of primary care access in Canada on supply/demand and structure/process domains.

This Perspective paper builds on prior isolated analyses of family physician shortage by integrating supply/demand, and structure/process frameworks to identify and discuss four complementary dimensions that can guide the analysis without presupposing a single cause (Figure 1).

These dimensions are intended to structure policy discussion and guide measurement for solutions that the public will recognize as improved access.

## 2 Dimensions

### 2.1 Effective capacity, not just headcount

Counting physicians is a weak proxy for the volume and continuity of care delivered. Over the last three decades, average weekly physician work hours in Canada declined by about 6.9 h—from 52.8 (1987–1991) to 45.9 (2017–2021)—with the decline concentrated among male physicians (2). Parallel analyses emphasize that planning should consider full-time equivalent (FTE) supply rather than absolute counts, because aging populations and changing hours both alter the true service capacity available (6).

Beyond hours, practice intensity has shifted. A cross-provincial study showed that while FP counts grew (e.g., Ontario + 35.3%; Alberta + 48.7% between 2005/06 and 2017/18), annual service days per FP declined (−10.6% Ontario; −5.9% Alberta) (7). Nationally, CIHI reports that the average number of patients seen per FP per year fell from 1,746 to 1,353 between 2013 and 2021 (3). Together these data indicate that time per patient, administrative load, and coordination demands are squeezing the number of encounters an FP can provide—reducing effective capacity even if headcount rises.

Crucially, “optimal” panel size is not a single magic number; it depends on continuity, team supports, patient complexity, and organizational model. Evidence from Ontario suggests nuanced relationships between panel size and quality, underscoring that policies to expand attachment must guard continuity and comprehensiveness rather than chase a uniform target (8).

### 2.2 Demand, complexity, time, and continuity

In primary care, what patients feel as “shortage” often reflects a dynamic gap: Δ*Demand exceeds* Δ*Effective supply*. Demand has grown along four fronts—demography, multimorbidity/complexity, *relational* demand for continuity, and non-visit tasks—while effective supply (clinician time that converts into longitudinal care) has not proportionally expanded.

Demography change and multimorbidity expand clinical time needs. Canada is aging: by 1 July 2023, 18.9% of the population was 65+, and the share will continue to rise (with very old age growing fastest) (9). Aging brings multimorbidity and polypharmacy that lengthen visits and coordination needs. A systematic review finds patients with multimorbidity require longer consultation times than those without, directly compressing the number of encounters a clinician can provide per day (10). CIHI’s newest international survey of older adults further shows lower timely access in Canada versus peers, consistent with demand outpacing available appointment capacity (11).

Continuity of care can also be viewed as relational demand, not just a quality metric. Patients do not only seek a slot; they seek *ongoing relationships* with a clinician/team that knows them. Evidence shows patients value continuity (e.g., seeing the same primary care doctor) for trust, not repeating histories, and comfort—and systems that deliver continuity achieve better reported experiences (12). Continuity is also outcome-relevant: a landmark systematic review across multiple settings associates higher continuity with lower mortality (13). When large numbers of people lack a regular provider—86% of adults had one in 2023 (down from 93% in 2016), leaving roughly 4 million without—*unmet relational demand* accumulates and is perceived as shortage, particularly among higher-need groups (1). Among older adults (65+), 8% still report no regular source of care—highest among 10 countries surveyed—illustrating how the continuity gap is itself part of demand (11).

Finally, complexity extends beyond diagnoses: tasks and coordination consume time. Administrative and coordination tasks (forms, information chasing, prior approvals, e-mails, inbox management) absorb millions of clinician hours annually in Canada, contributing to burnout and reducing effective clinical supply (14). Critical reviews catalog these burdens (compliance, learning, psychological costs) and their crowd-out of direct care (15). In short, even if headcount holds, *task inflation* increases demand on clinician time, narrowing appointment availability.

### 2.3 Maldistribution and loss of comprehensiveness

Another driver of “shortage” is *where* and *how* FPs practice. CIHI documents a marked shift of FPs toward focused roles outside traditional community-based comprehensive care. Nearly 30% of Canada’s FPs now practice predominantly outside primary care (e.g., emergency medicine or psychiatry), up from about 26% in 2019 to 28.3% in 2022 (3). This shift reduces the pool of clinicians available for longitudinal attachment and first-contact care, even if total FP numbers appear stable.

Population-level analyses from Ontario sharpen this picture. From 1993/94 to 2021/22, the proportion of FPs in focused practice rose from 7.7% to 19.2%, while the number of comprehensive FPs per 100,000 residents fell (16). More recently, growth in the *comprehensive* FP workforce stagnated post-2019 (9,377 in 2019 vs. 9,375 in 2022), with an increasing share near retirement; patients attached to near-retirement FPs are older and have higher chronic disease burdens, raising transition risks (17, 18).

Maldistribution also includes urban–rural and intra-urban patterns: growth in FP counts has tended to be greater in urban areas, while service days per FP have dropped across geographies, amplifying access gaps in smaller communities (7). Combined with fewer comprehensive FPs and rising patient complexity, these spatial and scope shifts *feel* like a “shortage” at the point of care—even where nominal FP supply has grown.

### 2.4 System entry point

In systems where primary care is the front door, how patients are attached, how they enter, and how teams are organized strongly shape experienced “shortage.” Canada continues to post the lowest same/next-day access and among the lowest after-hours access of 10 peer countries—signals of entry-point design as much as raw headcount (1). Countries with near-universal attachment typically combine enrolment/rostering with a clear general-practice “home,” robust after-hours arrangements, and interdisciplinary teams (5, 19).

Canada has piloted many of these organizational levers—but with uneven results. Ontario’s patient-enrolment models (e.g., blended capitation with formal rostering and after-hours obligations) and funded interprofessional teams transformed local primary care, yet early evaluations found mixed gains on access and attachment despite strong intent, underscoring that design details and implementation fidelity matter (20, 21). Meanwhile, at least six provinces have used centralized waiting lists to broker attachment for “orphan” patients; evaluations in Québec show CWLs can attach patients but performance varies by local design and resourcing (22–24).

Peer systems illustrate how entry-point architecture supports continuity and equity. Denmark’s list-based GP registration and gatekeeping model delivers high continuity and coordinated access across sectors—features repeatedly linked to better outcomes (25, 26). The lesson for Canada is not to copy-paste, but to commit to enrolment-first models with dependable after-hours access and team capacity, and to use CWLs as a bridge—not a substitute—for stable longitudinal attachment.

## 3 Policy solution discussion

Building on the four diagnostic dimensions, solutions can be placed in the following three categories: the system level, the organizational level, and data and research.

System-level change: At the system level, the first lever is to set—and protect—an explicit investment floor for primary care and tie new dollars to measurable improvements in attachment, same/next-day access, after-hours access,. and continuity OECD benchmarking shows primary health care averages roughly 13% of current health spending across member countries, and peer systems with stronger primary-care architecture achieve better attachment and timely access (5, 19). A second lever is to make enrolment/rostering the default point of entry so that demand flows through a longitudinal home rather than episodic settings; countries such as Denmark combine list-based registration, gatekeeping, and after-hours arrangements to channel urgent needs without fragmenting care (19, 27). Centralized Waiting Lists (CWLs) should be used as a bridge—not a substitute—for attachment, with design features (triage by complexity, real-time capacity, feedback loops) that evaluations have linked to better performance (23). Payment policy should align with this managed entry point: evidence from Ontario links funding models to variation in ambulatory-care-sensitive hospitalizations, and recent commentary argues that blended contracts should explicitly measure and reward continuity, after-hours access, and team-delivered comprehensiveness (28, 29). Finally, shrinking administrative drag is a system responsibility: standardized forms, delegated documentation, and EMR usability targets can reclaim clinician time and reduce burnout, converting funding into effective capacity (14).

Organizational-level change: Within organizations, interdisciplinary teams should be funded and staffed with clear roles, shared goals, and local implementation supports (leadership, co-location, panel management, and huddles). Systematic reviews and Canadian scoping reviews indicate that well-implemented teams improve access and comprehensiveness, although effects depend on design fidelity (30, 31). Task-sharing and clinical substitution are central: high-quality evidence shows that nurses (including nurse practitioners) can deliver care comparable to physicians for many conditions with equal or higher patient satisfaction (32). Pharmacist prescribing for minor ailments—now expanded in Ontario and being monitored at scale in British Columbia—has been found safe and effective and can offload low-acuity demand (33–35). Physician assistants also contribute meaningfully to first-contact capacity where funding rules and supervision are clear; Canadian data show high supervising-physician satisfaction but highlight the need for stable organizational funding (36). Crucially, continuity must be operationalized inside teams—e.g., a “named clinician” within a “named team,” explicit continuity metrics, and protected follow-up—because relational continuity is consistently associated with better outcomes, including lower mortality (13, 37).

Data and research: A data strategy should mirror the problem’s reframing and make indicator construction transparent. First, a pan-Canadian effective capacity dashboard should report not only headcount but effective FTE by geography and model (clinical hours, scope, panel complexity, and administrative time), while stating how each indicator is built. For example, CIHI’s “patients seen per family physician” is derived from National Physician Database billing/claims by fiscal year; it is not roster/panel size and can be influenced by alternative payment plans and shadow billing, with recent series excluding some jurisdictions (e.g., Québec, PEI, New Brunswick, territories), which limits comparability (3, 4). Estimates of physician hours commonly rely on Statistics Canada’s Labour Force Survey and reflect self-reported “usual weekly hours” across professional activities (clinical plus administrative), rather than direct patient-contact time (2). Second, analyses should attend to provincial heterogeneity: primary care reforms and workforce trends vary by jurisdiction; cross-provincial comparisons should present stratified estimates and document operational definitions (e.g., “service day” thresholds in claims-based studies) to support valid aggregation (7). Third, attachment and continuity should be core performance indicators, with routine public reporting of usual-provider continuity (UPC) and team continuity indices, given the consistent association between continuity and outcomes, including lower mortality (13, 37). Fourth, demand should be defined and weighted by tracking age structure, multimorbidity, and social complexity to create complexity-adjusted panel targets, reflecting evidence that multimorbidity lengthens consultations and compresses throughput (10). Fifth, entry-point reforms and scope expansions (enrolment/rostering with after-hours obligations; payment alignment; expanded roles for NPs, pharmacists, and PAs) should be evaluated with rigorous designs—stepped-wedge rollouts, difference-in-differences, and interrupted time series—with pre-specified outcomes (attachment, continuity, primary-care-sensitive ED use, ACSC hospitalizations, equity), noting current gaps where evidence remains heterogeneous or observational (28, 35). Finally, administrative burden should be measured and reduced via explicit provincial targets (form elimination, inbox delegation, EMR usability), with linkage to reclaimed clinical time, patients seen, and attachment gains (1, 14).

Implementing the proposed agenda will face predictable barriers. Governance and financing are fragmented across provinces and territories, creating variable readiness for enrolment and rostering, after-hours obligations, and payment alignment. Payment reform is path-dependent and may encounter stakeholder resistance such as concerns about risk selection, administrative load, or perceived loss of autonomy, unless continuity and equity safeguards are explicit. Scaling team-based care requires stable funding, role clarity, and workforce pipelines for NPs, pharmacists, and PAs, alongside regulatory alignment on scope of practice. Data infrastructure remains uneven: alternative payments and shadow billing complicate measurement; common definitions, privacy-respecting data linkages, and real-time evaluation capacity are prerequisites. Finally, rural and underserved settings will need tailored supports such as funding, recruitment incentives, virtual care and after-hours networks to avoid widening inequities during transition.

## 4 Conclusion

Canada’s primary care crisis is best understood not as a simple headcount gap but as the interaction of effective capacity, demand (complexity, time, continuity), maldistribution and loss of comprehensive care, and system entry-point design—the forces that produce what patients experience as “shortage.” Accordingly, solutions must align at three levels: system (adequate investment, managed enrolment, and incentives that reward continuity and team-delivered comprehensiveness), organizational (well-implemented interprofessional teams and task-sharing), and data/research (measuring effective FTE, attachment, continuity, and evaluating reforms rigorously). If governments, organizations, and researchers act on these levers together, Canadians should see tangible gains in timely attachment and relational continuity.

## Data Availability

The original contributions presented in this study are included in this article/supplementary material, further inquiries can be directed to the corresponding author.
